# Monitoring Access to Child Medicines: Introducing a Standardized Set of Age-Appropriate Medicines

**DOI:** 10.3390/children11030266

**Published:** 2024-02-20

**Authors:** Iris R. Joosse, Aukje K. Mantel-Teeuwisse, Fatima Suleman, Hendrika A. van den Ham

**Affiliations:** 1 Utrecht WHO Collaborating Centre for Pharmaceutical Policy and Regulation, Division of Pharmacoepidemiology and Clinical Pharmacology, Utrecht Institute for Pharmaceutical Sciences (UIPS), Utrecht University, Universiteitsweg 99, 3584 CG Utrecht, The Netherlands; i.r.joosse@uu.nl (I.R.J.); a.k.mantel@uu.nl (A.K.M.-T.); 2 WHO Collaborating Centre for Pharmaceutical Policy and Evidence Based Practice, School of Health Sciences, University of KwaZulu-Natal, Durban 4000, South Africa; sulemanf@ukzn.ac.za

**Keywords:** access to medicines, Sustainable Development Goals, primary health care, age-appropriate medicines, child health, Delphi technique

## Abstract

Monitoring access to pediatric medicines as part of the Sustainable Development Goal (SDG) agenda for 2030 requires surveying age-appropriate medicines. This study aimed to develop tracer sets of essential age-appropriate medicines for use in SDG indicator 3.b.3 or in conjunction with other methodologies for monitoring access to medicines. Two sets of medicines were developed, one for young children (1 month to 5 years) and one for school-aged children (5–12 years). Priority diseases were selected based on the global burden of disease and linked to active ingredients of first choice according to treatment guidelines and the World Health Organization (WHO) Model List of Essential Medicines for Children (EMLc). To ensure clinical relevance, the Delphi technique was employed to identify areas of (dis)agreement among clinical pediatric experts. During two consultation rounds, experts were invited to indicate (dis)agreement. Five experts per age group were largely in agreement with the initial selections, but various therapeutic alternatives were suggested for addition. A second consultation round with five experts did not lead to major adjustments. The final sets included 26 treatment options for both groups. Specific age-appropriate formulations were selected from the WHO EMLc 2023. These two globally representative tracer sets of medicines consider the particular needs of children and could aid countries in the critical monitoring of accessibility to pediatric medicines.

## 1. Introduction

The substantial number of preventable child deaths persists as a challenge in resource-limited countries [1]. Essential medicines are a crucial component in achieving further reductions in child mortality [2]. However, these medicines can only save lives when they are available, affordable, and acceptable to those who rely on them [3], which is frequently not the case [4]. As such, improving access to safe, effective, and quality-assured medicines for all is an important target within the Sustainable Development Goals (SDGs), embodied within targets 3.8 and 3.b of the SDGs [5]. SDG indicator 3.b.3—measuring the proportion of primary health facilities with a core set of relevant essential medicines available and affordable on a sustainable basis—was developed by the World Health Organization (WHO) to monitor countries’ current performance and track progress [6].

Although monitoring access to medicines is considered key to driving improvement and evaluating the impact of implemented solutions [7], SDG indicator 3.b.3 has significant limitations when applied to medicines for children [8]. These limitations stem from technical challenges in calculating affordability through the core metrics’ reliance on adult dosing schemes (i.e., Defined Daily Dosages, DDDs) and the core set of essential medicines as defined by the WHO being of limited relevance to children. This is evident from the small number of active ingredients that are a priority for children—given their propensity for different diseases compared to adults—and the lack of age-appropriate formulations in this core set. The latter is particularly relevant as children are a heterogeneous group requiring different dosages and with varying abilities to take medicines [9,10]. Specifically, children under five typically lack the ability to swallow solid oral dosage forms (i.e., conventional tablets, capsules), whereas the manipulation of medicines risks toxic or sub-therapeutic doses and dosing errors and may affect the stability of the product [11].

To allow monitoring of access to child-appropriate medicines through SDG indicator 3.b.3, we proposed and validated a methodology tailored to children, effectively addressing the technical challenges associated with calculating affordability [8,12]. However, as part of these adaptations, the child indicator—similar to the original indicator—requires the survey of a standardized set of tracer essential medicines [6]. No standardized set of medicines specifically tailored to the needs of children was available, as any existing sets were either outdated, merely reflected the health needs of a subgroup of children, or failed to include major therapeutic areas such as tuberculosis and HIV/AIDS [13,14]. As such, the aim of the present study was to develop a standardized tracer set of age-appropriate essential medicines—including medicines for both acute and chronic, as well as communicable and non-communicable diseases in the primary health care setting—representative for children of all ages.

## 2. Methods

Recognizing that children of different ages may require distinct dosage forms and strengths as well as other active ingredients, we created two core sets of medicines, one for children aged 1 month to 5 years (from now on referred to as young children) and one for children aged 5 to 12 years (from now on referred to as school-aged children).

### 2.1. Initial Selection of Core Set

Similar to the original methodology [6], the global burden of disease served as a foundation for identifying priority diseases in children. Ten diseases with the highest burden (and which can be managed with essential medicines as defined by the WHO in 2019 [15]) were selected based on Global Health Estimates (GHEs) for each age group [16]. *Pain and palliative care* is not reflected in the GHEs but was added separately as this is critical supportive care in many common conditions.

Priority diseases were then linked to essential medicines. As the core set is not meant to be a complete set of medicines but merely indicative of access, we limited ourselves to active ingredients of first choice in primary care that were also on the 7th WHO Model List of Essential Medicines for Children (EMLc, 2019). For young children, the WHO pocket book of hospital care for children represented the core reference [17]. As a similar (global) comprehensive guideline for children aged above 5 years was not available, the South African Standard Treatment Guidelines (STGs) for primary care were used [18]. This low- and middle-income country (LMIC) has well-established procedures for developing and updating their Standard Treatment Guidelines, and its STGs are representative of the diseases encountered and resources available in other LMICs. For disease areas not sufficiently described in either reference, relevant disease-specific treatment guidelines developed by the WHO were used [19,20,21,22,23,24]. These are global consensus guidelines targeted at LMICs, developed through a transparent, evidence-based decision-making process and subject to rigorous quality checking. If not available, other globally representative guidelines were selected [25,26].

### 2.2. Expert Consultation on Active Ingredients

To ensure that the primary selection sufficiently addressed the priority health needs in clinical practice, the Delphi technique was employed to identify areas of (dis)agreement among clinical experts. As an anonymous investigation method, the Delphi technique facilitates consensus building among geographically diverse experts through iterated rounds of structured data collection and controlled feedback to participants [27]. Participant expertise is critical to the validity of this technique. In this study, pediatricians or pharmacists specializing in pediatrics with at least 2 years of experience (to ensure sufficient experiential knowledge) were considered eligible to participate. To represent the global nature of SDG indicator 3.b.3, we recruited experts from all over the world and from different income levels. Participants were initially recruited from the 22nd WHO Expert Committee on Selection and Use of Essential Medicines. This was complemented with experts recruited through a network approach. Recruitment took place between March 2021 and February 2023 and stopped when the survey was completed by 5 experts per age group.

In an online survey (Annex S1), the background for the core set was described, and an explanation was given for the selection of disease areas. For each disease area, the experts were presented with the selected active ingredients and requested to indicate (dis)agreement with this selection. Participants were invited to provide an explanation if they indicated disagreement and specify any redundant medicines. Additionally, alternatives from the 7th WHO EMLc for the disease were presented, and experts were asked to indicate if there were any active ingredients missing from the selection. In the second part of the survey, experts were invited to indicate which specific formulation (dosage form and strength) from the 7th WHO EMLc they believed was preferred for each active ingredient in the primary selection for the respective age group.

The survey was piloted among 3 experts. This led to only minor refinements to the survey’s main part on active ingredient level, and the results were hence used in the final data analysis. Responses to the pilot questions on preferred formulations showed great variation in preferences between participants. From participants’ comments, it was deduced that their preference depended to a great extent on local availability of specific formulations. Due to these inconsistencies and in an effort to shorten the time needed to complete the survey, the questions on preferred formulations were eliminated from the final survey.

Online surveys were conducted using validated and password-protected software (LimeSurvey^®^ Version 4), and data were stored in line with legal requirements. Confidentiality of participants was maintained throughout the project.

In the data analysis, (dis)agreement on active ingredient level was assessed. Areas of (dis)agreement were identified following an 80% consensus rule, as follows: ≥80% of respondents indicating agreement was considered consensus; ≥80% of respondents indicating an active ingredient to be redundant was considered reason to remove the respective active ingredient. If no consensus was reached (<80% agreement) or when alternatives were suggested, the explanations provided were analyzed in depth to reach a decision. This involved comparing provided justifications against treatment guidelines, the 8th WHO EMLc (2021) [28], and the relevant literature. Since the initial core sets for the two age groups included many of the same active ingredients and disease areas, results were also cross-referenced between the two groups.

Upon completion of the data analysis, adjustments were made to the initial selection based on the experts’ input and in alignment with the latest available treatment guidelines [29,30,31]. In a second consolidation round, experts that had previously participated and indicated willingness to participate in a follow-up round were approached to take part in a second survey in April and May 2023. In the survey, experts were presented with the changes made to the initial selection and the arguments for these changes. If alternatives previously suggested had not been added to the selection, arguments were also provided. For each disease area, they were invited to indicate (dis)agreement. Due to the limited number of participants, all disagreements or comments were analyzed in depth to reach a final selection of active ingredients.

### 2.3. Selecting Child-Appropriate Formulations

Subsequently, specific formulations of active ingredients were selected because the availability of age-appropriate formulations is required for safe and effective treatment. As there was little to no agreement in preferred dosage forms and strengths between participants, age-appropriate formulations were thus selected pragmatically. This selection was based on formulations as listed on the 9th WHO EMLc (2023) [32], the doses required per age group, and practical assumptions (provided in Annex S2). For instance, we deemed it unreasonable if a child had to take more than two solid oral dosage units during an intake moment. Recommended (maintenance) doses per day in children—used for its main indication in primary healthcare—were determined based on international treatment guidelines or from the British National Formulary for Children (BNFC) if not specified in the respective guideline [33]. Weight-for-age charts were used to convert weight-based dosing to age-based dosing for the respective age groups [34,35,36]. Median weights of boys and girls within an age group were averaged to obtain a single measure per group. Medicine dosing based on body surface area was converted through an extra calculation step using the Meeh-type equation [37].

Formulations on the 9th WHO EMLc were then assessed for their appropriateness for the respective age group based on the required doses calculated and practical arguments (Annex S2). Multiple formulations of an active ingredient could be appropriate for a single age group to allow for variations in local market availability. Recommended doses were also used to estimate the number of units needed for treatment (NUNT), a child-specific parameter required to allow calculation of the indicator for children [8].

## 3. Results

A total of 11 priority disease areas were selected for each age group, with considerable overlap between age groups; diarrheal diseases, epilepsy, HIV/AIDS, iron-deficiency anemia, lower respiratory infections, malaria, meningitis, pain and palliative care, and tuberculosis were common across both groups. Measles and (congenital) syphilis were exclusively selected for young children, whereas asthma and migraine were unique for school-aged children. Upon examination of the treatment guidelines, 25 (combinations of) active ingredients (including therapeutic alternatives) for young children (Table 1) and 24 for school-aged children (Table 2) were selected. Some active ingredients were included in multiple disease areas.

Eight pediatricians and two pediatric pharmacists/pharmacologists participated in the first consultation round, amounting to five experts for each age group. Both of the pediatric pharmacists/pharmacologists were part of the school-aged group. The median years of experience were 17.5 (range: 7–40 years). Experts had collectively gained experience across 13 countries from all income levels and across four WHO regions (African Region, Region of the Americas, South-East Asian Region, and European Region; see Annex S3 for general characteristics of participants per age group).

Experts for both age groups were largely in agreement with the initial selection of active ingredients, with the exception of medicines for HIV/AIDS and lower respiratory infections (see Table 3). A considerable number of alternative active ingredients for addition to the core sets were suggested by the experts. Upon inspection of the justifications provided for young children and review of the relevant guidelines, chloramphenicol-based options were removed from the selection, four novel options were added (folic acid, hydroxocobalamin, benzathine penicillin, and antibiotics for treatment of dysentery/cholera), and five therapeutic alternatives were added. Additionally, the separate treatment options for HIV/AIDS in children under or above three years of age were combined under a single option. Other than the addition of two novel treatment options for migraine, changes to the core set for school-aged children were largely similar to those for young children. A detailed argumentation for the changes in the selection, including justifications for not incorporating alternatives proposed by the experts, can be found in Annex S4.

Nine experts had previously indicated being willing to participate in a follow-up round and were approached to take part again, of whom five completed the follow-up survey. These experts indicated overall agreement with the changes made to the core sets, with few areas of disagreement remaining (see Annex S4). Specifically, participant 7 remarked that diarrheal diseases seldom have a bacterial origin, and antibiotics would be irrational for this indication. Since four other participants indicated agreement with the addition of antibiotics, they were retained in the core set.

Nonetheless, doxycycline was removed as an alternative for young children as it is to be used in children under 8 years in exceptional circumstances only [32]. A single participant—participant 10—suggested that malaria treatment is situation-specific, and with increasing resistance to artemisinin-based combination therapies (ACTs), the addition of quinine should be reconsidered. Because the use of quinine is discouraged in the latest malaria treatment guidelines compared to ACTs [30], quinine was not added at this time. Two participants had reservations about the deletion of chloramphenicol combinations in the treatment of meningitis. Chloramphenicol had been removed out of an expert’s concern for toxicity, but participants 4 and 7 expressed that this concern may extend to other active ingredients within the core set as well and may therefore not be sufficient justification for deletion. Since chloramphenicol is specified as the second choice in bacterial meningitis on the WHO EMLc [32] and two other antibiotics are included in the core sets for this indication, we did not reintroduce it on the list at this time. Finally, participant number 7 also indicated disagreement with the addition of propranolol in the management of migraine, but no arguments were provided. The final selections can be found in Table 4 and Table 5.

For all active ingredients in the final selection, age-appropriate medicines were selected, and the number of units needed for a course treatment was determined. These can be found in Annex S5.

## 4. Discussion

To enable monitoring of access to medicines for children, this study proposes core sets of tracer medicines for two age groups with global implications and reflective of clinical practice. Although these tracer sets were primarily developed to enable monitoring of access to medicines for children as part of the SDG agenda, they hold broader relevance and can be used effectively in conjunction with other tools and methodologies such as the WHO/HAI methodology [38] and the forthcoming WHO Essential Medicines and Health Products Price and Availability Monitoring Mobile Application (MedMon) data collection tool [39]. The proposed core sets include medicines for the management of a range of childhood diseases, which are together representative of access to child medicines in a country. With that, these tracer sets indirectly also contribute to other targets on the SDG agenda, such as the reduction in under-five mortality (target 3.2) and the eradication of AIDS, tuberculosis, and malaria (target 3.3) [5]. To accommodate different national contexts, the proposed tracer sets offer several flexibilities through therapeutic alternatives and multiple acceptable formulations. The core sets for both age groups are largely similar in targeted disease areas and active ingredients, but critical differences arise from the selected age-appropriate formulations.

With the intention to encourage accountability at “the national, regional and global levels” and to “foster exchange of best practices and mutual learning”, the United Nations (UN) has committed to a systematic follow-up and review of agreed-upon goals and targets through indicators [40]. To promote such global benchmarking of SDG indicator 3.b.3, there is a need for a universal methodology and, by extension, a standardized set of medicines for comparison. At the same time, it is acknowledged that for these indicators to be impactful for individual member states, the indicators must be country context-specific [41]. These inherent opposites cause friction, necessitating concessions to balance global comparability and national applicability. In the case of indicator 3.b.3, this is a delicate balance between creating a core set with global relevance while also accommodating variations in local availability due to licensing and marketing differences, national best practices or guidelines, and local antimicrobial resistance patterns.

To foster global comparability of performance through international comparison, we utilized global tools as a foundation for the core sets. This involved global burden of disease estimates, complemented with treatment principles from widely accepted international treatment guidelines and selecting only WHO EMLc-listed medicines. Although medicines of local importance may have been missed through this approach, the core sets are not meant to be exhaustive but rather to function as a tracer set that is indicative of overall access for children. However, it is worth noting that the selection of priority diseases was based on disease burden (i.e., disability-adjusted life years) as opposed to disease prevalence. Childhood diseases with a high prevalence but low burden—such as eczema—may thus not be adequately captured. Similarly, global disease burden estimates for neonates are dominated by conditions such as preterm birth complications, birth asphyxia and birth trauma, neonatal sepsis and infections, and congenital anomalies [16]. These are associated with high mortality but are not representative of neonatal conditions managed at the primary care level [42]. For example, vitamin K-associated bleeding in neonates is not represented in the GHEs but is considered a standard of care for all newborns [43,44]. As systematic data on neonatal conditions managed in primary care are lacking, neonates were excluded from the present study. We highlight this as an important field of future study.

We have attempted to increase the national applicability of this indicator through several means. Firstly, insights from global pediatric experts were gathered to ensure that the tracer sets reflect their clinical practice. Moreover, possible acceptable alternatives were outlined in the core sets, granting countries the flexibility to select the most relevant active ingredients and formulations. Nonetheless, fundamental differences exist in disease burden across countries. This is particularly evident in the case of infectious diseases such as malaria, HIV/AIDS, and tuberculosis, of which the burden is relatively negligible in certain regions. SDG indicator 3.b.3 intends to correct for this by weighting according to the regional burden of diseases [6]. Whether this is the optimal approach to account for this remains to be determined [12]. Furthermore, the core sets comprise several medicines administered through injections. As the indicator targets primary healthcare facilities, it is important to note that in some countries, these facilities may not be equipped or authorized to administer injections. Additionally, given the continuously evolving clinical insights and the availability of new age-appropriate formulations and revisions to guidelines, a periodic review of the core sets is necessary. This will ensure that these core sets consistently reflect these dynamics.

Although the present study provides the tools to start monitoring access to child medicines as part of the Sustainable Development Goal agenda, actual monitoring of access to child medicines—or medicines in general—requires the deficiencies in data to be addressed urgently [45]. Monitoring access to medicines has previously failed as part of the Millennium Development Goals due to a lack of data [46], and this target was again omitted from the 2020 SDG progress report [47]. This data gap is not exclusive to indicator 3.b.3 [47] and calls for swift action from the international community to ensure that the important monitoring of SDGs can take place for all the indicators in the global indicator framework.

This study is subject to several limitations. Firstly, the recruitment of pediatric care experts was complicated by the COVID-19 pandemic. Recruitment was therefore delayed, and data were collected over a long period of time, primarily affecting the school-aged children group. This had some impact on the initial consultation round—with treatment guidelines getting updated in the meantime—but it is unlikely to have affected the final results of this study, with a second consultation round having taken place in 2023. Secondly, a small number of experts per age group took part in this study, with limited attrition of experts in the second round. However, considering that the questions in our survey were not open-ended but rather presented a predetermined selection of medicines to which the experts could indicate their agreement, the number of possible answers was restricted, and less variability in responses was expected. Additionally, to ensure content validity, we cross-checked results across age groups for analog disease areas and active ingredients. Thirdly, a pragmatic approach was used to select age-appropriate formulations for our survey based on those listed on the WHO EMLc—whose 2023 update included a review of the age-appropriate formulations on the list [48]—and international treatment guidelines. This selection could not be validated by clinical experts, as a pilot demonstrated that expert input was inconsistent. Finally, a few areas of disagreement remained after two consultation rounds with experts. These areas should be explored again in a periodic review of the core sets.

## 5. Conclusions

Monitoring progress is a core element of the SDG agenda for 2023 and key to achieving progress in access to age-appropriate medicines. This study introduces two globally representative tracer sets of medicines that consider the particular needs of children, allowing systematic monitoring of access to pediatric medicines as part of the SDG agenda for the first time. Beyond this, the tracer sets can be used in conjunction with other existing tools and methodologies for measuring access to medicines. While these tracer sets are fundamental to monitoring access to child medicines, concerted efforts are needed to address the existing data deficiencies. Only through parallel endeavors can we draw nearer to achieving access to medicines for all.

## Figures and Tables

**Table 1:** Initial and provisional selections of active ingredients for young children (aged 1 month to 5 years).

Initial Selection	Provisional Selection
**Diarrheal diseases**	
Oral rehydration salts	Oral rehydration salts
Zinc sulphate	Zinc sulphate
	**Doxycycline AND/OR ciprofloxacin OR azithromycin** **†**
**Epilepsy**	
Carbamazepine OR phenobarbital OR phenytoin	Carbamazepine OR phenobarbital OR phenytoin
Valproic acid	Valproic acid **OR lamotrigine**
Diazepam OR lorazepam OR midazolam	Diazepam OR lorazepam OR midazolam
**HIV/AIDS**	
*Children <3 years:* Abacavir + lamivudine + lopinavir/ritonavir OR zidovudine + lamivudine + lopinavir/ritonavir OR abacavir + lamivudine + nevirapine OR zidovudine + lamivudine + nevirapine	*Children 1 month–5 years*: **Abacavir + lamivudine + dolutegravir** OR abacavir + lamivudine + lopinavir/ritonavir
*Children 3–5 years:* Abacavir + lamivudine + efavirenz OR abacavir + lamivudine + nevirapine OR zidovudine + lamivudine + efavirenz OR zidovudine + lamivudine + nevirapine	
**Anemia**	
Ferrous salt	Ferrous salt
Mebendazole OR albendazole	Mebendazole OR albendazole
	**Folic acid**
	**Hydroxocobalamin**
**Lower respiratory infections**	
Amoxicillin	Amoxicillin **OR amoxicillin + clavulanic acid**
Ampicillin	Ampicillin
Benzylpenicillin	Benzylpenicillin **OR phenoxymethylpenicillin**
Gentamicin	Gentamicin
Ceftriaxone	Ceftriaxone **OR cefotaxime**
**Malaria**	
Artemether + lumefantrine OR artesunate + amodiaquine OR artesunate + mefloquine OR dihydroartemisinin + piperaquine OR artesunate + sulfadoxine-pyrimethamine	Artemether + lumefantrine OR artesunate + amodiaquine OR artesunate + mefloquine OR dihydroartemisinin + piperaquine OR artesunate + sulfadoxine-pyrimethamine **OR artesunate + pyronaridine**
Artesunate	Artesunate
**Measles**	
Retinol	Retinol
**Meningitis**	
Ceftriaxone	Ceftriaxone
Cefotaxime	Cefotaxime
Chloramphenicol + ampicillin	
Chloramphenicol + benzylpenicillin	
**Pain and palliative care**	
Paracetamol	Paracetamol
Ibuprofen	Ibuprofen
Morphine	Morphine
**(Congenital) syphilis**	
Benzylpenicillin	Benzylpenicillin **OR** procaine benzylpenicillin
Procaine benzylpenicillin	**Benzathine penicillin**
**Tuberculosis**	
Ethambutol + isoniazid + pyrazinamide + rifampicin	Ethambutol + isoniazid + pyrazinamide + rifampicin

† Doxycycline or ciprofloxacin or azithromycin are appropriate choices for treatment of cholera. If doxycycline is selected for survey, either ciprofloxacin or azithromycin should also be added for treatment of dysentery. 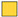 (alternative) active ingredient added to selection; 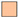 active ingredient removed.

**Table 2:** Initial and provisional selections of active ingredients for school-aged children (aged 5 to 12 years).

Initial Selection	Provisional Selection
**Asthma**	
Salbutamol	Salbutamol
Budesonide	Budesonide
**Diarrheal diseases**	
Oral rehydration salts	Oral rehydration salts
Zinc sulphate	Zinc sulphate
	**Doxycycline AND/OR ciprofloxacin OR azithromycin** **†**
**Epilepsy**	
Carbamazepine OR phenobarbital OR phenytoin	Carbamazepine OR phenobarbital OR phenytoin
Valproic acid	Valproic acid **OR lamotrigine**
Diazepam OR lorazepam OR midazolam	Diazepam OR lorazepam OR midazolam
**HIV/AIDS**	
Abacavir + lamivudine + efavirenz OR abacavir + lamivudine + nevirapine OR zidovudine + lamivudine + efavirenz OR zidovudine + lamivudine + nevirapine	**Abacavir + lamivudine + dolutegravir** OR abacavir + lamivudine + lopinavir/ritonavir
**Anemia**	
Ferrous salt	Ferrous salt
Albendazole	**Mebendazole** OR albendazole
	**Folic acid**
	**Hydroxocobalamin**
**Lower respiratory infections**	
Amoxicillin	Amoxicillin **OR amoxicillin + clavulanic acid**
Ampicillin	Ampicillin
Benzylpenicillin	Benzylpenicillin OR **phenoxymethylpenicillin**
Gentamicin	Gentamicin
Ceftriaxone	Ceftriaxone OR **cefotaxime**
**Malaria**	
Artemether + lumefantrine OR artesunate + amodiaquine OR artesunate + mefloquine OR dihydroartemisinin + piperaquine OR artesunate + sulfadoxine-pyrimethamine	Artemether + lumefantrine OR artesunate + amodiaquine OR artesunate + mefloquine OR dihydroartemisinin + piperaquine OR artesunate + sulfadoxine-pyrimethamine OR **artesunate + pyronaridine**
Artesunate	Artesunate
**Meningitis**	
Ceftriaxone	Ceftriaxone
Cefotaxime	Cefotaxime
Chloramphenicol + ampicillin	
Chloramphenicol + benzylpenicillin	
**Migraine**	
Ibuprofen	Ibuprofen
	**Paracetamol**
	**Propranolol**
**Pain and palliative care**	
Paracetamol	Paracetamol
Ibuprofen	Ibuprofen
Morphine	Morphine
**Tuberculosis**	
Ethambutol + isoniazid + pyrazinamide + rifampicin	Ethambutol + isoniazid + pyrazinamide + rifampicin

† Doxycycline or ciprofloxacin or azithromycin are appropriate choices for treatment of cholera. If doxycycline is selected for survey, either ciprofloxacin or azithromycin should also be added for treatment of dysentery. 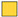 (alternative) active ingredient added to selection; 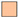 active ingredient removed.

**Table 3:** Agreement of experts with initial selection of active ingredients and number of experts suggesting alternatives per disease area.

	Agreement with Presented Selection										Alternatives Suggested by (n)	
	Young					School-Aged					Young	School-Aged
Participant No.	1	2	3	4	5	6	7	8	9	10		
Asthma											-	1
Diarrheal diseases											1	1
Epilepsy											2	2
HIV/AIDS (<3 years)											3	-
HIV/AIDS (>3 years)											3	2
Iron-deficiency anemia											0	4
Lower respiratory infections											2	3
Malaria											2	2
Measles											0	-
Meningitis											1	1
Migraine											-	4
Pain and palliative care											0	0
Syphilis (congenital)											1	-
Tuberculosis											3	0

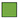
 in agreement with selection; 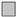 not completed by participant; 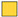 disagreement with selection; 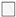 not applicable to age group.

**Table 4:** Final selection of (combinations of) active ingredients for survey per disease area for young children (1 month–5 years).

No.	(Combinations of) Active Ingredients for Survey	GHE Code
	**Anemia**	**580 + 590**
1	Ferrous salt	
2	Mebendazole OR albendazole	
3	Folic acid	
4	Hydroxocobalamin	
	**Diarrheal diseases**	**110**
5	Oral rehydration salts	
6	Zinc sulphate	
7	Ciprofloxacin OR azithromycin	
	**Epilepsy**	**970**
8	Carbamazepine OR phenobarbital OR phenytoin	
9	Valproic acid OR lamotrigine	
10	Diazepam OR lorazepam OR midazolam	
	**HIV/AIDS**	**100**
11	Abacavir + lamivudine + dolutegravir OR abacavir + lamivudine + lopinavir/ritonavir	
	**Lower respiratory infections**	**370 + 390**
12	Amoxicillin OR amoxicillin + clavulanic acid	
13	Ampicillin	
14	Benzylpenicillin OR phenoxymethylpenicillin	
15	Gentamicin	
16 ^a^	Ceftriaxone OR cefotaxime	
	**Malaria**	**220**
17	Artemether + lumefantrine OR artesunate + amodiaquine OR artesunate + mefloquine OR dihydroartemisinin + piperaquine OR artesunate + sulfadoxine-pyrimethamine OR artesunate + pyronaridine	
18	Artesunate	
	**Measles**	**150 + 570**
19	Retinol	
	**Meningitis**	**170 + 370**
20 ^a^	Ceftriaxone	
21 ^a^	Cefotaxime	
	**Pain and palliative care**	**-**
22	Paracetamol	
23	Ibuprofen	
24	Morphine	
	**(Congenital) syphilis**	**50**
25	Benzylpenicillin OR procaine benzylpenicillin	
26	Benzathine penicillin	
	**Tuberculosis**	**30**
27	Ethambutol + isoniazid + pyrazinamide + rifampicin	

One active ingredient (or combination of active ingredients) must be selected per number (No.). A total of 26 active ingredients (or combinations of active ingredients) are selected. Associated GHE codes are used when assigning weights according to burden of disease in calculating SDG indicator 3.b.3. GHE = Global Health Estimates. Pain and palliative care is not associated with a GHE code. ^a^ Ceftriaxone and cefotaxime are included for two indications.

**Table 5:** Final selection of (combinations of) active ingredients for survey per disease area for school-aged children (5–12 years).

No.	Final Selection	GHE Code
	**Anemia**	**580 + 590**
1	Ferrous salt	
2	Mebendazole OR albendazole	
3	Folic acid	
4	Hydroxocobalamin	
	**Asthma**	**1190**
5	Salbutamol	
6	Budesonide	
	**Diarrheal diseases**	**110**
7	Oral rehydration salts	
8	Zinc sulphate	
9 ^a^	Doxycycline AND/OR ciprofloxacin OR azithromycin	
	**Epilepsy**	**970**
10	Carbamazepine OR phenobarbital OR phenytoin	
11	Valproic acid OR lamotrigine	
12	Diazepam OR lorazepam OR midazolam	
	**HIV/AIDS**	**100**
13	Abacavir + lamivudine + dolutegravir OR abacavir + lamivudine + lopinavir/ritonavir	
	**Lower respiratory infections**	**390 + 370**
14	Amoxicillin OR amoxicillin + clavulanic acid	
15	Ampicillin	
16	Benzylpenicillin OR phenoxymethylpenicillin	
17	Gentamicin	
18 ^b^	Ceftriaxone OR cefotaxime	
	**Malaria**	**220**
19	Artemether + lumefantrine OR artesunate + amodiaquine OR artesunate + mefloquine OR dihydroartemisinin + piperaquine OR artesunate + sulfadoxine-pyrimethamine OR artesunate + pyronaridine	
20	Artesunate	
	**Meningitis**	**170 + 370**
21 ^b^	Ceftriaxone	
22 ^b^	Cefotaxime	
	**Migraine**	**990**
23 ^c^	Ibuprofen	
24 ^c^	Paracetamol	
25	Propranolol	
	**Pain and palliative care**	**-**
26 ^c^	Paracetamol	
27 ^c^	Ibuprofen	
28	Morphine	
	**Tuberculosis**	**30**
29	Ethambutol + isoniazid + pyrazinamide + rifampicin	

One active ingredient (or combination of active ingredients) must be selected per number (No.). A total of 26 (or 27 if doxycycline is selected) active ingredients (or combinations of active ingredients) are selected. Associated GHE codes are used when assigning weights according to burden of disease in calculating SDG indicator 3.b.3. Pain and palliative care is not associated with a GHE code. GHE = Global Health Estimates. ^a^ Doxycycline or ciprofloxacin or azithromycin are appropriate choices for treatment of cholera. If doxycycline is selected for survey, either ciprofloxacin or azithromycin should also be added for treatment of dysentery. ^b^ Ceftriaxone and cefotaxime are included for two indications. ^c^ Ibuprofen and paracetamol are included for two indications.

## Data Availability

The data presented in this study are available on request from the corresponding author. The data are not publicly available due to privacy reasons.

## Supplementary Materials

The following supporting information can be downloaded at: https://www.mdpi.com/article/10.3390/children11030266/s1, Annex S1: Survey questions for round 1; Annex S2: Pragmatic assumptions in selecting age-appropriate formulations; Annex S3: General characteristics of participants; Annex S4: Justifications for (un)alterations in initial core sets of medicines and results of consolidation round with experts; Annex S5: Age-appropriate formulations and associated number of units needed for treatment.
