# Supplementary material for: Monitoring Access to Child Medicines: Introducing a Standardized Set of Age-Appropriate Medicines
Source: Children (Basel). 2024 Feb 20;11(3):266. doi: 10.3390/children11030266 (PMC10969351; doi:10.3390/children11030266)
Supplement: Supplementary file 1 [file children-11-00266-s001.zip › children-2840639-supplementary.pdf]

Annex S1

Survey questions for round 1

These surveys were conducted online with LimeSurvey®.

Young children

Title: A methodology for measuring access to medicines for children

Description: With this questionnaire, we will validate the selection of a core set of essential medicines for young children.

WELCOME TEXT

Dear participant, thank you for helping us by filling in this questionnaire.

This questionnaire is part of a project called 'A methodology for measuring access to essential medicines for children'. You are being asked to take part in this research project because you are an expert on the use of medicines in neonates or children. The questionnaire will take approximately 25 minutes of your time.

In case of questions, problems or remarks, you may contact Iris Joosse: [*email*].

INFORMED CONSENT

I have been invited to participate in a research project, titled "Methodology for measuring access to essential medicines for children". I am being asked to take part in this project because I am an expert on the use of medicines in neonates or children.

I have read the information. I have had the opportunity to ask questions about it and any questions I have asked, have been answered to my satisfaction. I consent voluntarily to be a participant in this study. I am aware that I can withdraw at any point in time without negative consequences, and without providing any explanation.

I am aware that participation in this study is confidential. I consent to the use of my personal data in line with legal requirements such as the Data Protection Fundamental Directive in force in the EU for all EU personal data. I give permission for storing the research data for a period of ten years.

☐ I consent to participate in this study

IDENTIFYING INFORMATION

First name(s):

Last name:

Institution:

Position:

Expertise: (Choose pediatrician OR neonatologist OR specialized pharmacist)

Country:

Email contact:

INTRODUCTION

A key element in achieving universal health coverage (UHC) is the provision of access to medicines for all, as described in target 3.b. of the Sustainable Development Goals (SDGs)[1]. To monitor countries' performance and progress on improving access to medicines, SDG indicator 3.b.3. has been developed (formula 1)[2]:

$$SDG_{3.b.3.} = \frac{Facilities\ with\ available\ and\ affordable\ basket\ of\ medicines\ (n)}{Surveyed\ Facilities\ (n)} \quad (1)$$

The indicator includes three core concepts used for calculating indicator 3.b.3:

1) A core set of globally relevant essential medicines
2) Availability of medicines
3) Affordability of medicines

However, SDG indicator 3.b.3 has been developed and piloted for measuring access to medicines in general and is predominantly targeted at adults. The methodology as has been developed for adults does not necessarily apply to pediatric medicines. Having an indicator that can reflect the situation for children is essential, especially in the Sub-Saharan setting where a large part of the population consists of children under the age of fifteen.

Objectives

In the present study, we are adapting the original SDG indicator 3.b.3. computation methodology so that it can be used to calculate access to medicines for children. As part of creating a standardized methodology for measuring access to medicines for children, we have defined a core set of globally relevant essential (pediatric) medicines, that are indicative of the access to medicines in primary health care. The goal of this questionnaire is to validate our selection of medicines for young children with your expertise.

---

[1] United Nations. Sustainable Development Goals Knowledge Platform. URL: sustainabledevelopment.un.org/?menu=1300.
[2] United Nations Statistics Division. SDG Indicators Metadata repository. 2019. URL: https://unstats.un.org/sdgs/metadata?Text=&Goal=3&Target=3.b.

VALIDATING THE SELECTION OF ACTIVE INGREDIENTS

The core set of essential medicines for children addresses the health needs of children for a variety of globally prevalent childhood diseases. Priority medicines for each disease are selected from the WHO model List of Essential Medicines for children (EMLc) 2019. Selection of active ingredients was based on three criteria:

1) Active ingredients are first-choice medicines, according to international treatment guidelines[3];
2) Active ingredients are not vaccines or non-pharmaceutical products (e.g. medicinal gas);
3) Cold chain management is not required for the active ingredient.

In this part of the questionnaire, we present our selection of active ingredients for young children. We ask you to indicate if you agree with our selection and if any active ingredients are redundant or missing.

Note: the core set of globally relevant essential medicines should be **indicative** of access to medicines for the majority of children/cases. It is **not** exhaustive or meant to cover all cases.

START SURVEY

In the tables below, the selection of active ingredients for eleven priority diseases in young children is shown. Column 1 shows the selection of active ingredients as made by the researchers. Column 2 shows alternative active ingredients included on the Essential Medicines List for Children (EMLc), that can be used to treat the condition.

Diarrhoeal diseases

**Selected first-choice medicines**	**Alternative medicines on EMLc**
Oral rehydration salts<br>Zinc sulphate	Sulfamethoxazole + trimethoprim<br>Azithromycin<br>Cefotaxime<br>Ceftriaxone<br>Ciprofloxacin

1. Do you agree with the selection of active ingredients for diarrhoeal diseases?
   Yes
   No → If no, please indicate which medicine(s) is/are redundant and why.

2. In your opinion, are there any other medicines from the second column that should be considered essential first-choice? You may choose alternative active ingredients not shown above.
   Yes → If yes, please indicate which and why.
   No

Epilepsy

**Selected first-choice medicines**	**Alternative medicines on EMLc**
Carbamazapine or phenobarbital or phenytoin<br>Valproic acid<br>Diazepam or lorazepam or midazolam	Lamotrigine

[3] WHO treatment guidelines were adhered to, if available.

3. Do you agree with the selection of active ingredients for epilepsy?
   Yes
   No → If no, please indicate which medicine(s) is/are redundant and why.

4. In your opinion, are there any other medicines from the second column that should be considered essential first-choice? You may choose alternative active ingredients not shown above.
   Yes → If yes, please indicate which and why.
   No

HIV/AIDS

**Selected first-choice regimens**	**Alternative medicines on EMLc**
Children < 3 years	
Abacavir + lamivudine + lopinavir/ritonavir OR<br>Zidovudine + lamivudine + lopinavir/ritonavir OR<br>Abacavir + lamivudine + nevirapine OR<br>Zidovudine + lamivudine + nevirapine	Ritonavir<br>Raltegravir
Children >3 years	
Abacavir + lamivudine + efivarenz OR<br>Abacavir + lamivudine + nevirapine OR<br>Zidovudine + lamivudine + efivarenz OR<br>Zidovudine + lamivudine + nevirapine	Atazanavir<br>Darunavir<br>Ritonavir<br>Dolutegravir<br>Raltegravir

5. Do you agree with the selection of active ingredients for HIV/AIDS for children <3 years?
   Yes
   No → If no, please indicate which medicine(s) is/are redundant and why.

6. In your opinion, are there any other medicines from the second column that should be considered essential first-choice in children <3 years? You may choose alternative active ingredients not shown above.
   Yes → If yes, please indicate which and why.
   No
7. Do you agree with the selection of active ingredients for HIV/AIDS for children >3 years?
   Yes
   No → If no, please indicate which medicine(s) is/are redundant and why.

8. In your opinion, are there any other medicines from the second column that should be considered essential first-choice in children >3 years? You may choose alternative active ingredients not shown above.
   Yes → If yes, please indicate which and why.
   No

Iron-deficiency anemia

**Selected first-choice medicines**	**Alternative medicines on EMLc**
Ferrous salt<br>Mebendazole or albendazole	Folic acid<br>Hydroxocobalamin

9. Do you agree with the selection of active ingredients for iron-deficiency anemia?

Yes
No → If no, please indicate which medicine(s) is/are redundant and why.

10. In your opinion, are there any other medicines from the second column that should be considered essential first-choice? You may choose alternative active ingredients not shown above.
Yes → If yes, please indicate which and why.
No

Lower respiratory infections

**Selected first-choice regimens**	**Alternative medicines on EMLc**
Amoxicillin<br>Ampicillin<br>Benzylpenicillin<br>Gentamicin<br>Ceftriaxone	Amoxicillin + clavulanic acid<br>Phenoxymethylpenicillin<br>Cefotaxime<br>Piperacillin + tazobactam

11. Do you agree with the selection of active ingredients for lower respiratory infections?
Yes
No → If no, please indicate which medicine(s) is/are redundant and why.

12. In your opinion, are there any other medicines from the second column that should be considered essential first-choice? You may choose alternative active ingredients not shown above.
Yes → If yes, please indicate which and why.
No

Malaria

**Selected first-choice medicines**	**Alternative medicines on EMLc**
Artemether + lumefantrine<br>Artesunate + amodiaquine<br>Artesunate + mefloquine<br>Dihydroartemisinin + piperaquine<br>Artesunate + Sulfadoxine-pyrimethamine<br>Artesunate	Artesunate + pyronaridine tetraphosphate<br>Chloroquine<br>Doxycycline<br>Primaquine<br>Quinine

13. Do you agree with the selection of active ingredients for malaria?
Yes
No → If no, please indicate which medicine(s) is/are redundant and why.

14. In your opinion, are there any other medicines from the second column that should be considered essential first-choice? You may choose alternative active ingredients not shown above.
Yes → If yes, please indicate which and why.
No

Measles

**Selected first-choice medicines**	**Alternative medicines on EMLc**

Retinol	X

15. Do you agree with the selection of active ingredients for measles?
    Yes
    No → If no, please indicate which medicine(s) is/are redundant and why.

16. In your opinion, are there any other medicines from the second column that should be considered essential first-choice? You may choose alternative active ingredients not shown above.
    Yes → If yes, please indicate which and why.
    No

Meningitis

Selected first-choice medicines	Alternative medicines on EMLc
Ceftriaxone<br>Cefotaxime<br>Chloramphenicol + ampicillin<br>Chloramphenicol + benzylpenicillin	Amoxicillin<br>Meropenem

17. Do you agree with the selection of active ingredients for meningitis?
    Yes
    No → If no, please indicate which medicine(s) is/are redundant and why.

18. In your opinion, are there any other medicines from the second column that should be considered essential first-choice? You may choose alternative active ingredients not shown above.
    Yes → If yes, please indicate which and why.
    No

Pain and palliative care

Selected first-choice medicines	Alternative medicines on EMLc
Paracetamol<br>Ibuprofen<br>Morphine	Methadone

19. Do you agree with the selection of active ingredients for pain and palliative care?
    Yes
    No → If no, please indicate which medicine(s) is/are redundant and why.

20. In your opinion, are there any other medicines from the second column that should be considered essential first-choice? You may choose alternative active ingredients not shown above.
    Yes → If yes, please indicate which and why.
    No

(Congenital) syphilis

Selected first-choice medicines	Alternative medicines on EMLc
Benzylpenicillin	Benzathine benzylpenicillin

Procaine benzylpenicillin	

21. Do you agree with the selection of active ingredients for syphilis?
    Yes
    No → If no, please indicate which medicine(s) is/are redundant and why.

22. In your opinion, are there any other medicines from the second column that should be considered essential first-choice? You may choose alternative active ingredients <u>not</u> shown above.
    Yes → If yes, please indicate which and why.
    No

Tuberculosis

**Selected first-choice medicines**	**Alternative medicines on EMLc**
Ethambutol + isoniazid + pyrazinamide + rifampicin	Rifapentine

23. Do you agree with the selection of active ingredients for tuberculosis?
    Yes
    No → If no, please indicate which medicine(s) is/are redundant and why.

24. In your opinion, are there any other medicines from the second column that should be considered essential first-choice? You may choose alternative active ingredients <u>not</u> shown above.
    Yes → If yes, please indicate which and why.
    No

Final questions

	**Yes**	**No**
25. I give permission to be acknowledged by name in the final publication of this study	☐	☐
26. I want to receive the final results of this study	☐	☐
27. If consensus is not reached after the first round of surveys, the study personnel may contact me for participation in an online focus group:	☐	☐

Thank you for your participation.

School-aged children

Title: A methodology for measuring access to medicines for children

Description: With this questionnaire, we will validate the selection of a core set of essential medicines for school-aged children.

WELCOME TEXT

Dear participant, thank you for helping us by filling in this questionnaire.

This questionnaire is part of a project called ‘A methodology for measuring access to essential medicines for children’. You are being asked to take part in this research project because you are an expert on the use of medicines in neonates or children. The questionnaire will take approximately 25 minutes of your time.

In case of questions, problems or remarks, you may contact Iris Joosse: [*email*].

INFORMED CONSENT

I have been invited to participate in a research project, titled "Methodology for measuring access to essential medicines for children”. I am being asked to take part in this project because I am an expert on the use of medicines in neonates or children.

I have read the information. I have had the opportunity to ask questions about it and any questions I have asked, have been answered to my satisfaction. I consent voluntarily to be a participant in this study. I am aware that I can withdraw at any point in time without negative consequences, and without providing any explanation.

I am aware that participation in this study is confidential. I consent to the use of my personal data in line with legal requirements such as the Data Protection Fundamental Directive in force in the EU for all EU personal data. I give permission for storing the research data for a period of ten years.

☐ I consent to participate in this study

IDENTIFYING INFORMATION

First name(s):

Last name:

Institution:

Position:

Expertise: (Choose pediatrician OR neonatologist OR specialized pharmacist)

Country:

Email contact:

INTRODUCTION

A key element in achieving universal health coverage (UHC) is the provision of access to medicines for all, as described in target 3.b. of the Sustainable Development Goals (SDGs)[4]. To monitor countries' performance and progress on improving access to medicines, SDG indicator 3.b.3. has been developed (formula 1)[5]:

$$SDG_{3.b.3.} = \frac{Facilities\ with\ available\ and\ affordable\ basket\ of\ medicines\ (n)}{Surveyed\ Facilities\ (n)} \quad (1)$$

The indicator includes three core concepts used for calculating indicator 3.b.3:

1) A core set of globally relevant essential medicines
2) Availability of medicines
3) Affordability of medicines

However, SDG indicator 3.b.3 has been developed and piloted for measuring access to medicines in general and is predominantly targeted at adults. The methodology as has been developed for adults does not necessarily apply to pediatric medicines. Having an indicator that can reflect the situation for children is essential, especially in the Sub-Saharan setting where a large part of the population consists of children under the age of fifteen.

Objectives

In the present study, we are adapting the original SDG indicator 3.b.3. computation methodology so that it can be used to calculate access to medicines for children. As part of creating a standardized methodology for measuring access to medicines for children, we have defined a core set of globally relevant essential (pediatric) medicines, that are indicative of the access to medicines in primary health care. The goal of this questionnaire is to validate our selection of medicines for school-aged children with your expertise.

---

[4] United Nations. Sustainable Development Goals Knowledge Platform. URL: sustainabledevelopment.un.org/?menu=1300.

[5] United Nations Statistics Division. SDG Indicators Metadata repository. 2019. URL: https://unstats.un.org/sdgs/metadata?Text=&Goal=3&Target=3.b.

VALIDATING THE SELECTION OF ACTIVE INGREDIENTS

The core set of essential medicines for children addresses the health needs of children for a variety of globally prevalent childhood diseases. Priority medicines for each disease are selected from the WHO model List of Essential Medicines for children (EMLc) 2019. Selection of active ingredients was based on three criteria:

1) Active ingredients are first-choice medicines, according to international treatment guidelines[6];
2) Active ingredients are not vaccines or non-pharmaceutical products (e.g. medicinal gas);
3) Cold chain management is not required for the active ingredient.

In this part of the questionnaire, we present our selection of active ingredients for school-aged children. We ask you to indicate if you agree with our selection and if any active ingredients are redundant or missing.

Note: the core set of globally relevant essential medicines should be **indicative** of access to medicines for the majority of children/cases. It is **not** exhaustive or meant to cover all cases.

START OF QUESTIONS

In the tables below, the selection of active ingredients for eleven priority diseases in school-aged children is shown. Column 1 shows the selection of active ingredients as made by the researchers. Column 2 shows alternative active ingredients included on the Essential Medicines List for Children (EMLc), that can be used to treat the condition.

Asthma

**Selected first-choice medicines**	**Alternative medicines on EMLc**
Salbutamol or other short-acting beta-agonist inhaler<br>Budesonide or other corticosteroid inhaler	Epinephrine (adrenaline)

28. Do you agree with the selection of active ingredients for asthma?
    Yes
    No, because…

29. In your opinion, are any alternative medicines that are on the EMLc missing from the selection?
    Yes, because…
    No

Diarrhoeal diseases

Selected first-choice medicines	Alternative medicines on EMLc
Oral rehydration salts<br>Zinc sulphate	Sulfamethoxazole + trimethoprim<br>Azithromycin<br>Cefotaxime<br>Ceftriaxone<br>Ciprofloxacin

30. Do you agree with the selection of active ingredients for diarrhoeal diseases?
Yes

[6] WHO treatment guidelines were adhered to, if available.

No, because…

31. In your opinion, are any alternative medicines that are on the EMLc missing from the selection?
    Yes, because…
    No

Epilepsy

Selected first-choice medicines	Alternative medicines on EMLc
Carbamazapine or phenobarbital or phenytoin<br>Valproic acid<br>Diazepam or lorazepam or midazolam	Lamotrigine

32. Do you agree with the selection of active ingredients for epilepsy?

Yes

No, because…

33. In your opinion, are any alternative medicines that are on the EMLc missing from the selection?
    Yes, because…
    No

HIV/AIDS

**Selected first-choice regimens**	**Alternative medicines on EMLc**
Abacavir + lamivudine + efivarenz<br>Abacavir + lamivudine + nevirapine<br>Zidovudine + lamivudine + efivarenz<br>Zidovudine + lamivudine + nevirapine	Atazanavir<br>Darunavir<br>Ritonavir<br>Dolutegravir<br>Raltegravir

34. Do you agree with the selection of active ingredients for HIV/AIDS?
    Yes
    No, because…

35. In your opinion, are any alternative medicines that are on the EMLc missing from the selection?
    Yes, because…
    No

Iron-deficiency anemia

Selected first-choice medicines	Alternative medicines on EMLc
Ferrous salt<br>Albendazole	Folic acid<br>Hydroxocobalamin

36. Do you agree with the selection of active ingredients for iron-deficiency anemia?

Yes

No, because…

37. In your opinion, are any alternative medicines that are on the EMLc missing from the selection?
    Yes, because…
    No

Lower respiratory infections

Selected first-choice regimens	Alternative medicines on EMLc
Amoxicillin<br>Ampicillin<br>Benzylpenicillin<br>Gentamicin<br>Ceftriaxone	Amoxicillin + clavulanic acid<br>Doxycycline<br>Phenoxymethylpenicillin<br>Cefotaxime<br>Piperacillin + tazobactam

38. Do you agree with the selection of active ingredients for lower respiratory infections?

Yes

No, because…

39. In your opinion, are any alternative medicines that are on the EMLc missing from the selection?
    Yes, because…
    No

Malaria

Selected first-choice medicines	Alternative medicines on EMLc
Artemether + lumefantrine<br>Artesunate + amodiaquine<br>Artesunate + mefloquine<br>Dihydroartemisinin + piperaquine<br>Artesunate + Sulfadoxine-pyrimethamine<br>Artesunate	Artesunate + pyronaridine tetraphosphate<br>Chloroquine<br>Doxycycline<br>Primaquine<br>Quinine

40. Do you agree with the selection of active ingredients for malaria?

Yes

No, because…

41. In your opinion, are any alternative medicines that are on the EMLc missing from the selection?
    Yes, because…
    No

Meningitis

Selected first-choice medicines	Alternative medicines on EMLc
Ceftriaxone<br>Cefotaxime<br>Chloramphenicol + ampicillin<br>Chloramphenicol + benzylpenicillin	Amoxicillin<br>Meropenem

42. Do you agree with the selection of active ingredients for meningitis?

Yes

No, because…

43. In your opinion, are any alternative medicines missing from the selection?
    Yes, because…
    No

Migraine

Selected first-choice medicines	Alternative medicines on EMLc
Ibuprofen	Paracetamol<br>Propranolol

44. Do you agree with the selection of active ingredients for migraine?
    Yes
    No, because…

45. In your opinion, are any alternative medicines that are on the EMLc missing from the selection?
    Yes, because…
    No

Pain and palliative care

Selected first-choice medicines	Alternative medicines on EMLc
Paracetamol<br>Ibuprofen<br>Morphine	Methadone

46. Do you agree with the selection of active ingredients for pain and palliative care?

Yes

No, because…

47. In your opinion, are any alternative medicines that are on the EMLc missing from the selection?
    Yes, because…
    No

Tuberculosis

Selected first-choice medicines	Alternative medicines on EMLc
Ethambutol + isoniazid + pyrazinamide + rifampicin	Rifapentine

48. Do you agree with the selection of active ingredients for tuberculosis?

Yes

No, because…

49. In your opinion, are any alternative medicines that are on the EMLc missing from the selection?
    Yes, because…
    No

FINAL QUESTIONS

	Yes	No
50. I give permission to be acknowledged by name in the final publication of this study	☐	☐
51. I want to receive the final results of this study	☐	☐
52. If consensus is not reached after the first round of surveys, the study personnel may contact me for participation in an online focus group:	☐	☐

Thank you for your participation.

Annex S2

Pragmatic assumptions in selecting age-appropriate formulations

As the group represents a range of ages and weights, required doses were also calculated for the smallest and biggest child in the respective age group. These lower and upper acceptable limits were used to make an approximation of appropriate dosing and the number of required units for a course treatment (number of units needed for treatment, NUNT). If there are no alternative dosage forms on the WHO EMLc, the respective dosage form is automatically considered appropriate.

Table S1 Pragmatic assumptions in selecting age-appropriate formulations

Dosage form	Assumptions
Solid oral dosage forms	Solid oral dosage forms cannot be split†.
	A minimum of 1 and a maximum of 2 units are administered per recommended intake moment.
Oral liquids	A minimum of 0.5 and a maximum of 10 ml are administered per recommended intake moment.
	Required millilitres (between 1-10 ml) are rounded to whole numbers.
Rectal dosage forms	Administration of a single unit only.
	Rectal dosage forms cannot be split.
Parenteral dosage forms	If oral or rectal dosage forms are available on the WHO EMLc, they are given preference over parenteral dosage forms.
	At least 1 vial/ampoule is used per day. A vial/ampoule can be used for multiple intake moments on the same day.

†Although splitting may be an appropriate alternative in individual cases or when no other dosage form is available, it is not the preferred option as manipulation risks administrating toxic or sub-therapeutic doses through inaccurate dosing, as well as dosing errors

Annex S3

General characteristics of participants

Table S2 general characteristics of participants

	Young children (n)	School-aged children (n)
**Expertise**		
Paediatrician	5	3
Paediatric pharmacist/ pharmacologist	0	2
**WHO region where experience was gained**		
African region	3	3
Region of the Americas	1	0
South-East Asian region	0	2
European region	2	1
**World Bank income classification†**		
Low-income country	3	1
Lower-middle income country	1	4
Upper-middle income country	2	0
High-income country	3	1

Experts could have gained experience across multiple countries. †Income classification 2022-2023

Annex S4

Justifications in (un)alterations in initial core sets of medicines and results of consolidation round with experts

The tables below provide justification for alterations and unalterations made to the initial selections of active ingredients, as also provided to experts in the consolidation round.

Table S3 Justifications for (un)alterations in initial selection for young children

Disease area	Arguments for (un)alternations in initial selection for young children
**Diarrheal diseases**	To address prevalent diarrheal diseases with a bacterial origin (e.g. cholera, dysentery), several **antibacterial** options were added to the selection.
**Epilepsy**	**Lamotrigine** was added as an alternative to valproic acid, particularly for refractory epilepsy and in case of drug interactions.<br>Although **levetiracetam** may be essential in specific populations, this medicine is currently not included in the WHO Model List of Essential Medicines for Children (one of our selection criteria). It is therefore also not included in the present selection.
**HIV/AIDS**	The initial selection was changed to reflect the first-choice medicines as described in the 2021 updated WHO HIV/AIDS guidelines. A separate basket for children under and over 3 years of age is no longer required.
**Anaemia**	To address anaemias other than iron-deficiency anaemia only, **folic acid** and **hydroxocobalamin** were added to the selection.
**Lower respiratory infections**	**Amoxicillin-clavulanic acid** was added as alternative to amoxicillin alone, as it may provide wider coverage.<br>**Phenoxymethylpenicillin** was added as an oral alternative to **benzylpenicillin**.<br>**Cefotaxime** was added as alternative to **ceftriaxone** for several reasons, because it only requires once-daily dosing and it may be a more affordable option. Additionally, there is registered microbial resistance to ceftriaxone.
**Malaria**	A sixth **ACT** alternative was added to reflect the 2022 updates to the WHO Malaria guidelines.<br>**Quinine** was not added as its use is discouraged compared to ACTs in recent treatment guidelines.<br>**Primaquine** is appropriate for use in specific cases only, and is therefore not considered one of the core medicines in malaria.
**Meningitis**	**Chloramphenicol** combinations were removed due to toxicity reports.
**Congenital syphilis**	**Benzylpenicillin** and **procaine benzylpenicillin** are now considered alternatives.<br>**Benzathine penicillin** was added for treating asymptomatic syphilis.

ACT = Artemisinin-based combination therapy

Table S4 Justifications for (non) changes in initial selection for school-aged children

Disease area	Arguments for (un)alterations in initial selection for school-aged children
**Diarrheal diseases**	To address prevalent diarrheal diseases with a bacterial origin (e.g. cholera, dysentery), several **antibacterial** options were added to the selection.
**Epilepsy**	**Lamotrigine** was added as an alternative to valproic acid, particularly for refractory epilepsy and in case of drug interactions.<br>Although **levetiracetam** may be essential in specific populations, this medicine is currently not included in the WHO Model List of Essential Medicines for Children (one of our selection criteria). It is therefore also not included in the present selection.
**HIV/AIDS**	The initial selection was changed to reflect the first-choice medicines as described in the 2021 updated WHO HIV/AIDS guidelines, and those available on the WHO EMLc 2021
**Anaemia**	To address anaemias other than iron-deficiency anaemia only, **folic acid** and **hydroxocobalamin** were added to the selection.

	**Mebendazole** was added as an alternative to albendazole.
**Lower respiratory infections**	**Amoxicillin-clavulanic acid** was added as alternative to amoxicillin alone, as it may provide wider coverage.<br>**Phenoxymethylpenicillin** was added as an oral alternative to **benzylpenicillin**.<br>**Cefotaxime** was added as alternative to **ceftriaxone** for several reasons, because it only requires once-daily dosing and it may be a more affordable option. Additionally, there is registered microbial resistance to ceftriaxone.
**Malaria**	A sixth **ACT** alternative was added to reflect the 2022 updates to the WHO Malaria guidelines.<br>**Quinine** was not added as its use is discouraged compared to ACTs in recent treatment guidelines.<br>**Primaquine** is appropriate for use in specific cases only, and is therefore not considered one of the core medicines in malaria.
**Meningitis**	**Chloramphenicol** combinations were removed due to toxicity reports.
**Migraine**	**Paracetamol** was added to the selection for acute migraine attacks, **propranolol** for potential prophylaxis.

ACT = Artemisinin-based combination therapy

Table S5 Agreement of experts with changes made to the selection of active ingredients

**Disease area**	**Active ingredient**	**Type of change**	**Young**		**School-aged**		
		Participant no.	2	4	6	7	10
Diarrheal diseases	Doxycycline, ciprofloxacin and azithromycin	Added to the selection (as alternatives)	in agreement	in agreement	in agreement	disagreement	in agreement
Epilepsy	Lamotrigine	Added to the selection as alternative	in agreement	not completed	in agreement	in agreement	in agreement
	Levetiracetam	Expert suggestion not adopted	in agreement	not completed	in agreement	in agreement	in agreement
HIV/AIDS	Abacavir + lamivudine + dolutegravir and abacavir + lamivudine + lopinavir/ritonavir	Replacing previous selection	in agreement	in agreement	in agreement	in agreement	in agreement
Anaemia	Folic acid	Added to the selection	in agreement	not completed	in agreement	in agreement	in agreement
	Hydroxocobalamin	Added to the selection	in agreement	not completed	in agreement	in agreement	in agreement
	Mebendazole	Added to the selection as alternative	not applicable	not applicable	in agreement	in agreement	in agreement
LRI	Amoxicillin-clavulanic acid	Added to the selection	in agreement	in agreement	in agreement	in agreement	in agreement
	Phenoxymethylpenicillin	Added to the selection	in agreement	in agreement	in agreement	in agreement	in agreement
	Cefotaxime	Added to the selection	in agreement	in agreement	in agreement	in agreement	in agreement
Malaria	Artesunate-pyronaridine	Added to the selection	in agreement	in agreement	in agreement	in agreement	in agreement
	Quinine	Expert suggestion not adopted	in agreement	in agreement	in agreement	in agreement	disagreement
	Primaquine	Expert suggestion not adopted	in agreement	in agreement	in agreement	in agreement	in agreement
Meningitis	Chloramphenicol	Removed from selection	in agreement	disagreement	in agreement	disagreement	in agreement
Migraine	Paracetamol	Added to the selection	not applicable	not applicable	in agreement	in agreement	in agreement
	Propranolol	Added to the selection	not applicable	not applicable	in agreement	disagreement	in agreement
Congenital syphilis	Benzylpenicillin and procaine benzylpenicillin	Combined as alternatives	in agreement	in agreement	not applicable	not applicable	not applicable
	Benzathine penicillin	Added to the selection	in agreement	in agreement	not applicable	not applicable	not applicable

in agreement with change(s)
disagreement with change(s)
not completed by participant
not applicable to age group

LRI = lower respiratory infections

Annex S5

Age-appropriate formulations and associated number of units needed for treatment

Table S6 Appropriate formulations and associated number of units needed for treatment for young children (1 month-5 years)

Active ingredient	Appropriate formulations	NUNT
Abacavir/lamivudine	Tablet (dispersible, scored) 120/60 mg	60 units
Albendazole	Tablet (chewable, scored) 400 mg	1 unit
Amoxicillin	Powder for oral liquid 125 mg/5 ml	100 ml
	Powder for oral liquid 250 mg/5 ml	90 ml
	Solid oral dosage form 250 mg	20 units
	Solid oral dosage form 500 mg	10 units
	Tablet (dispersible, scored) 250 mg	20 units
	Tablet (dispersible, scored) 500 mg	10 units
Amoxicillin/clavulanic acid	Powder for oral liquid 125/31.25 mg/5 ml	100 ml
	Powder for oral liquid 250/62.5 mg/5 ml	90 ml
	Tablet 500/125 mg	10 units
	Tablet (dispersible) 200/28.5 mg	20 units
	Tablet (dispersible) 250/62.5 mg	20 units
Ampicillin	Powder for injection 500 mg in vial	20 vials
Artemether/lumefantrine	Tablet 20/120 mg	6 units
	Tablet (dispersible) 20/120 mg	6 units
Artesunate	Injection, ampoule containing 60 mg	1 ampoule
	Rectal dosage form 50 mg	1 unit
	Tablet 50 mg†	3 units
Artesunate/amodiaquine	Tablet 25/67.5 mg	6 units
	Tablet 50/135 mg	3 units
Artesunate/mefloquine	Tablet 25/55	6 units
Artesunate/pyronaridine	Granules 20/60 mg	6 sachets
Azithromycin	Solid oral dosage form 250 mg	1 unit
	Powder for oral liquid 200 mg/5 ml	6 ml
Benzathine penicillin	Powder for injection 1.2 million IU (900 mg) in vial	1 vial
Benzylpenicillin	Powder for injection 600 mg (1 million IU) in vial	20 vials
	Powder for injection 3 g (5 million IU) in vials	10 vials
Carbamazepine	Oral liquid 100 mg/5 ml	240 ml
	Tablet (chewable) 100 mg	60 units
	Tablet (chewable) 200 mg	30 units
	Tablet (scored) 100 mg	60 units
	Tablet (scored) 200 mg	30 units
Cefotaxime	Powder for injection 500 mg in vial	28 vials
	Powder for injection 1 g in vial	14 vials
Ceftriaxone	Powder for injection 500 mg in vial	14 vials
	Powder for injection 1 g in vial	7 vials
Ciprofloxacin	Oral liquid 250 mg/5 ml	18 ml
	Solid oral dosage form 100 mg	12 units
	Solid oral dosage form 250 mg	6 units
Diazepam	Rectal gel 5 mg/ml in 0.5 ml rectal delivery system	1 tube
	Rectal solution 2 mg/ml in 1.25 ml rectal tube	1 tube
	Rectal solution 2 mg/ml in 2.5 ml rectal tube	1 tube
Dihydroartemisinin/piperaquine	Tablet 20/160 mg	6 units
	Tablet 40/320 mg	3 units
Dolutegravir	Tablet (dispersible, scored) 10 mg	60 units
Ethambutol	Tablet 100 mg	60 units
	Tablet (dispersible) 100 mg	60 units
Ferrous salt	Oral liquid equivalent to 25 mg iron/ml	90 ml

Folic acid	Tablet 1 mg	30 units
	Tablet 5 mg	30 units
Gentamicin	Injection 40 mg/ml in 2 ml vial	5 vials
Hydroxocobalamin	Injection 1 mg in 1 ml ampoule	4 ampoules
Ibuprofen	Oral liquid 100 mg/5 ml	480 ml
	Oral liquid 200 mg/5 ml	240 ml
Isoniazid	Tablet 100 mg	30 units
	Tablet (dispersible) 100 mg	30 units
Isoniazid/pyrazinamide/rifampicin‡	Tablet (dispersible) 50/150/75 mg	60 units
Isoniazid/rifampicin‡	Tablet (dispersible) 50/75 mg	60 units
Lamotrigine	Tablet 25 mg	60 units
	Tablet 50 mg	30 units
	Tablet 100 mg	30 units
	Tablet (chewable, dispersible) 25 mg	60 units
	Tablet (chewable, dispersible) 50 mg	30 units
	Tablet (chewable, dispersible) 100 mg	30 units
Lopinavir/ritonavir	Tablet (heat stable) 100/25 mg	60 units
Lorazepam	Injection 2 mg/ml in 1 ml ampoule	1 ampoule
Mebendazole	Tablet (chewable) 100 mg	6 units
	Tablet (chewable) 500 mg	1 unit
Midazolam	Solution for oromucosal administration 5 mg/ml in 1 ml pre-filled syringe	1 syringe
	Solution for oromucosal administration 5 mg/ml in 1.5 ml pre-filled syringe	1 syringe
	Solution for oromucosal administration 10 mg/ml in 0.5 ml pre-filled syringe	1 syringe
	Solution for oromucosal administration 10 mg/ml in 0.75 ml pre-filled syringe	1 syringe
	Injection 1 mg/ml in 5 ml vial*	1 vial
	Injection 5 mg/ml in 1 ml vial*	1 vial
Morphine	Granules (slow release) 20 mg	30 sachets
	Oral liquid 10 mg/5 ml	180 ml
	Tablet (slow release) 10 mg	30 units
	Tablet (slow release) 20 mg	30 units
Oral rehydration salts (ORS)	Powder for dilution in 200 ml	5 sachets
	Powder for dilution in 500 ml	2 sachets
	Powder for dilution in 1 L	1 sachet
Phenobarbital	Oral liquid 15 mg/5 ml	300 ml
Paracetamol	Oral liquid 120 mg/5 ml or 125 mg/5 ml	840 ml
	250 mg/5 ml	360 ml
	Suppository 100 mg	120 units
	Tablet (dispersible) 10 mg	240 units
	Tablet 15 mg	120 units
	Tablet 30 mg	60 units
	Tablet 60 mg	30 units
	Tablet 100 mg	30 units
Phenoxymethylpenicillin	Powder for oral liquid 250 mg/5 ml	60 ml
	Solid oral dosage form 250 mg	20 units
Phenytoin	Oral liquid 30 mg/5 ml	420 ml
	Solid oral dosage form 25 mg	90 units
	Solid oral dosage form 50 mg	60 units
	Tablet (chewable) 50 mg	60 units
Procaine benzylpenicillin	Powder for injection 1 g (1 million IU) in vial	10 vials
Pyrazinamide	Tablet 400 mg	30 units
	Tablet 500 mg	30 units
Retinol	Capsule 100 000 IU	4 units

	Capsule 200 000 IU	2 units
	Oral oily solution in multidose dispenser	2 ml
Rifampicin	Oral liquid 20 mg/ml	240 ml
	Solid oral dosage form 150 mg	30 units
Sulfadoxine-pyrimethamine†	Tablet 500/25 mg	1 unit
Valproic acid	Oral liquid 200 mg/5 ml	300 ml
	Tablet (crushable) 100 mg	120 units
	Tablet (enteric-coated) 200 mg	60 units
Zinc sulphate	Solid oral dosage form 20 mg	14 units

For each active ingredient (or combination of active ingredients) selected in main text table 4 an appropriate formulation must be selected for survey. Flexible oral dosage forms (chewable, dispersible or scored tablets) should be given priority, if registered in the country. NUNT = number of units needed for treatment.

*For buccal administration when solutions are not available.

†Only in combination with artesunate 50 mg tablet.

‡Only in combination with ethambutol (and pyrazinamide). If fixed-dose combinations are not selected, all four active ingredients must be surveyed separately.

Table S7 Appropriate formulations and associated number of units needed for treatment for school-aged children (5-12 years)

Active ingredient	Appropriate formulations	NUNT
Abacavir/lamivudine	Tablet (scored, dispersible) 120/60 mg	90 units
Albendazole	Tablet (chewable, scored) 400 mg	1 unit
Amoxicillin	Solid oral dosage form 500 mg	20 units
	Tablet (dispersible, scored) 500 mg	20 units
Amoxicillin/clavulanic acid	Tablet 500/125 mg	20 units
Ampicillin	Powder for injection 1 g in vial	20 vials
Artemether/lumefantrine	Tablet 20/120 mg	12 units
	Tablet (dispersible) 20/120 mg	12 units
Artesunate	Injection, ampoule containing 60 mg	1 ampoule
	Rectal dosage form 100 mg	1 unit
	Tablet 50 mg†	6 units
Artesunate/amodiaquine	Tablet 50/135 mg	6 units
	Tablet 100/270 mg	3 units
Artesunate/mefloquine	Tablet 100/220	3 units
Artesunate/pyronaridine	Tablet 60/180	6 units
Azithromycin	Solid oral dosage form 250 mg	2 units
	Solid oral dosage form 500 mg	1 unit
Benzylpenicillin	Powder for injection 600 mg (1 million IU) in vial	40 vials
	Powder for injection 3 g (5 million IU) in vial	10 vials
Budesonide	Inhalation (aerosol) 100 mcg per dose	60 doses
	Inhalation (aerosol) 200 mcg per dose	30 doses
Carbamazepine	Tablet (chewable) 200 mg	60 units
	Tablet (scored) 200 mg	30 units
	Tablet (scored) 400 mg	30 units
Cefotaxime	Powder for injection 1 g in vial	14 vials
	Powder for injection 2 g in vial	14 vials
Ceftriaxone	Powder for injection 1 g in vial	14 vials
Ciprofloxacin	Oral liquid 250 mg/5 ml	48 ml
Diazepam	Rectal gel 5 ml/ml in 2 ml rectal delivery system	1 tube
	Rectal solution 4 mg/ml in 2.5 ml rectal tube	1 tube
Dihydroartemisinin/piperaquine	Tablet 40/320 mg	6 units
Dolutegravir	Tablet 50 mg	30 units
Doxycycline	Oral liquid 50 mg/5 ml	8 ml
	Solid oral dosage form 50 mg	2 units
	Solid oral dosage form 100 mg	1 unit
	Tablet (dispersible) 100 mg	1 unit
Ethambutol	Tablet 400 mg	30 units
Ferrous salt	Oral liquid equivalent to 25 mg iron/ml	150 ml
	Tablet equivalent to 60 mg iron	60 units
Folic acid	Tablet 5 mg	30 units
Gentamicin	Injection 40 mg/ml in 2 ml vial	10
Hydroxocobalamin	Injection 1 mg in 1 ml ampoule	4 ampoules
Ibuprofen	Oral liquid 100 mg/5 ml	1080 ml
	Oral liquid 200 mg/5 ml	600 ml
	Tablet 200 mg	120 units
Isoniazid	Tablet 100 mg	60 units
	Tablet 300 mg	30 units
	Tablet (dispersible) 100 mg	60 units
Lamotrigine	Tablet 50 mg	60 units
	Tablet 100 mg	30 units
	Tablet 200 mg	30 units
	Tablet (chewable, dispersible) 50 mg	60 units

	Tablet (chewable, dispersible) 100 mg	30 units
	Tablet (chewable, dispersible) 200 mg	30 units
Lopinavir/ritonavir	Tablet (heat stable) 100/25 mg	120 units
Lorazepam	Injection 2 mg/ml in 1 ml ampoule	1 ampoule
Mebendazole	Tablet (chewable) 500 mg	1 unit
Midazolam	Solution for oromucosal administration 5 mg/ml in 2 ml pre-filled syringe	1 syringe
	Solution for oromucosal administration 10 mg/ml in 1 ml pre-filled syringe	1 syringe
	Injection 5 mg/ml in 3 ml vial*	1 vial
Morphine	Granules (slow release) 20 mg	30 sachets
	Granules (slow release) 30 mg	30 sachets
	Oral liquid 10 mg/5 ml	540 ml
	Tablet (slow release)	90 units
	Tablet (slow release)	60 units
	Tablet (slow release)	30 units
Oral rehydration salts	Powder for dilution 200 ml	10 sachets
	Powder for dilution 500 ml	4 sachets
	Powder for dilution 1 L	2 sachets
Paracetamol	Oral liquid 250 mg/5 ml	960 ml
	Tablet 250 mg	120 units
	Tablet 325 mg	120 units
	Tablet (dispersible) 250 mg	120 units
Phenobarbital	Tablet 30 mg	120 units
	Tablet 60 mg	60 units
	Tablet 100 mg	30 units
Phenoxymethylpenicillin	Powder for oral liquid 250 mg/5 ml	60 ml
	Solid oral dosage form 250 mg	20 units
Phenytoin	Oral liquid 30 mg/5 ml	600 ml
	Solid oral dosage form 50 mg	120 units
	Solid oral dosage form 100 mg	60 units
	Tablet (chewable) 50 mg	120 units
Propranolol	Tablet 20 mg	60 units
Pyrazinamide	Tablet 400 mg	60 units
	Tablet 500 mg	60 units
Rifampicin	Solid oral dosage form 150 mg	60 units
	Solid oral dosage form 300 mg	30 units
Salbutamol	Metered dose inhaler (aerosol) 100 mcg	180 doses
Sulfadoxine-pyrimethamine†	Tablet 500/25 mg	1 unit
Valproic acid	Oral liquid 200 mg/5 ml	600 ml
	Tablet (enteric-coated) 200 mg	120 units
	Tablet (enteric-coated) 500 mg	60 units
Zinc sulphate	Solid oral dosage form 20 mg	14 units

For each active ingredient (or combination of active ingredients) selected in main text table 5 an appropriate formulation must be selected for survey. Flexible oral dosage forms (chewable, dispersible or scored tablets) should be given priority, if registered in the country. NUNT = number of units needed for treatment.

*For buccal administration when solutions are not available.

†Only in combination with artesunate 50 mg tablet.
